# Evidence of natural selection in the mitochondrial-derived peptides humanin and SHLP6

**DOI:** 10.1038/s41598-023-41053-0

**Published:** 2023-08-29

**Authors:** James M. Gruschus, Daniel L. Morris, Nico Tjandra

**Affiliations:** Laboratory of Structural Biophysics, Biochemistry and Biophysics Center, NHLBI, NIH, 50 South Drive, Bethesda, MD 20892 USA

**Keywords:** Evolutionary genetics, Insulin signalling, Cell signalling, Mitochondria, Apoptosis

## Abstract

Mitochondrial-derived peptides are encoded by mitochondrial DNA but have biological activity outside mitochondria. Eight of these are encoded by sequences within the mitochondrial 12S and 16S ribosomal genes: humanin, MOTS-c, and the six SHLP peptides, SHLP1-SHLP6. These peptides have various effects in cell culture and animal models, affecting neuroprotection, insulin sensitivity, and apoptosis, and some are secreted, potentially having extracellular signaling roles. However, except for humanin, their importance in normal cell function is unknown. To gauge their importance, their coding sequences in vertebrates have been analyzed for synonymous codon bias. Because they lie in RNA genes, such bias should only occur if their amino acids have been conserved to maintain biological function. Humanin and SHLP6 show strong synonymous codon bias and sequence conservation. In contrast, SHLP1, SHLP2, SHLP3, and SHLP5 show no significant bias and are poorly conserved. MOTS-c and SHLP4 also lack significant bias, but contain highly conserved N-terminal regions, and their biological importance cannot be ruled out. An additional potential mitochondrial-derived peptide sequence was discovered preceding SHLP2, named SHLP2b, which also contains a highly conserved N-terminal region with synonymous codon bias.

## Introduction

In this paper we evaluate the notion that peptides encoded by short open reading frames (ORFs) necessarily have important biological functions^1^. In particular, we focus on the mitochondrial-derived peptides (MDPs), of which humanin is the most well-known. When humanin was discovered over twenty years ago, it was the first of its kind, a peptide encoded by mitochondrial DNA but with biological activity outside the mitochondrion. It was found in a screen for neuroprotective peptides using a cDNA library created from the surviving neurons of an Alzheimer’s disease patient^2^. Prior to this, the mitochondrial genome was thought to only encode 13 proteins, which all remain inside the mitochondria. However, humanin was found to inhibit apoptosis, interacting with the pro-apoptosis proteins BAX, BIM, BID and IGFBP-3 in the cell cytosol^5–8^. Furthermore, secreted humanin was detected in cell culture medium and extracellular humanin was found to interact with cell-surface receptor proteins FPRL-1 and CNTFR/WSX-1/gp130, which are involved in metabolism and inflammation signaling^2,9,10^. Interestingly, its anti-apoptotic property can also potentially be detrimental, as beyond a certain threshold, inhibiting cell death can promote tumor growth in cancer^11–13^.

**Figure 1:**
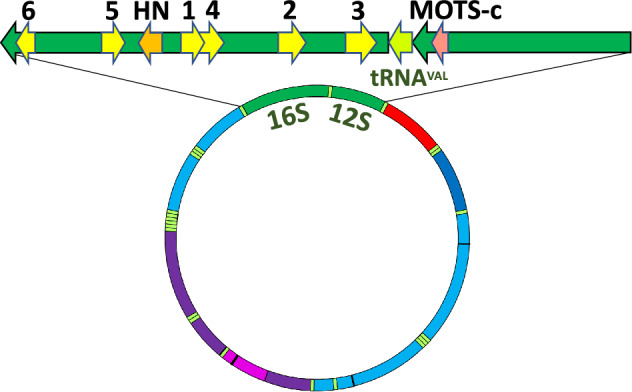
Location of MDPs in mtDNA. The locations of the MDP coding sequences in the 16S and 12S RNA genes (green) is indicated, with numbers indicating the SHLP peptides, SHLP1-SHLP6, and humanin (HN) and MOTS-c indicated. The arrows indicate coding by the mtDNA H-strand (pointing left) or L-strand (pointing right). Below, a diagram of the full circular mtDNA is shown for comparison. The tRNA sequences are in light green, the D-loop in red, and the protein genes in shades of blue and purple.

Humanin is unique in another way; it is encoded by a nested, overlapping gene within an RNA gene. The sequence encoding humanin lies within *MT-RNR2*, the gene for the 16S subunit of the mitochondrial ribosome. Recent searches of the mitochondrial 16S and 12S RNA genes have identified seven additional MDPs with potential biological activity. These are the six SHLPs (Small Humanin-Like Peptides), SHLP1-SHLP6, found within *MT-RNR2,* and MOTS-c (Mitochondrial Open-reading-frame of the Twelve S rRNA type-c), within the mitochondrial 12S RNA gene *MT-RNR1*^14,15^. A diagram showing the locations of their ORFs within the 16S and 12S RNA genes is shown in Fig. 1. Several potential biological activities for the SHLP peptides have been observed, including enhancement of cell survival by SHLP2 and SHLP3, cell proliferation by SHLP4, and induction of apoptosis by SHLP6^14^. MOTS-c production is increased by exercise, and treatment with exogenous peptide confers many of the same benefits as exercise in cell and animal models, including enhancement of insulin sensitivity^15,16^.

To demonstrate a peptide is biologically important, one can show that lowering its amount leads to dysfunction in the cell. While there is currently no way to knock out mitochondrial ORFs, they can be knocked down with siRNA (small interfering RNA). This has been done for humanin, where knockdown reduced its anti-apoptotic and neuroprotective effects^5,17^. However, such results for the other MDPs have not been forthcoming. One can also show the importance of a peptide using a more straightforward test which relies on evolution. Natural selection will conserve the amino acids important for the function of the peptide, so one can look for sequence conservation across species. Unfortunately, because the coding sequences of the MDPs lie within ribosomal genes, sequence conservation alone is insufficient as it might instead reflect the importance of the corresponding RNA bases for ribosome function. A complementary approach is to look for synonymous codons in the peptide-coding sequences. Like amino acid sequence conservation, synonymous codon bias is evidence of purifying, or negative, natural selection that has been “weeding out” non-synonymous amino acid mutations over the course of evolution. On the other hand, for non-peptide-coding regions of the RNA genes, there should be no bias. Thus, in this study, in addition to looking for sequence conservation, we examine synonymous codon bias in the MDP coding sequences. In doing so, not only do we determine which MDPs have been conserved by evolution but also identify shared features that offer clues to their biological roles.

## Results

### Humanin is conserved across vertebrates with many residues showing synonymous codon bias

**Figure 2:**
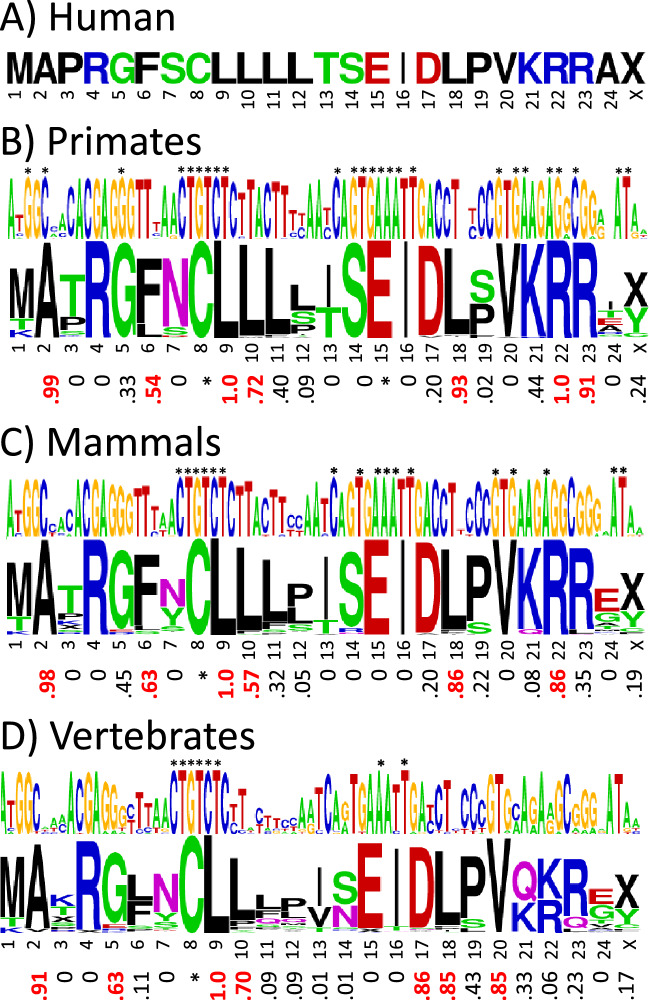
Synonymous codon bias in humanin. (**A**) Humanin sequence in humans. Sequence logos of the base and amino acid sequence alignments with the synonymous codon bias *f*_syn_ below in (**B**) primates (number of species = 252), (**C**) mammals (number of species = 148), and (**D**) vertebrates (number of species = 359). The base and amino acid letter heights indicate how conserved they are across species. Invariant bases and amino acids with invariant codons are indicated with asterisks. The “X” symbol stands for stop codon. Values of *f*_syn_ ≥ 0.5 are highlighted in red. Because methionine has only one codon in the standard DNA code, it has no *f*_syn_ value. The color code for the amino acids is hydrophobic (black), acidic (red), basic (blue), and neutral hydrophilic (green for glycine and hydroxyl-containing residues and magenta for amide-containing).

Figure 2 shows the consensus base and amino acid sequence logos for the primate, mammal, and vertebrate humanin alignments, with the corresponding *f*_syn_ values below. Amino acid positions with *f*_syn_ ≥ 0.5, that is, with equal or more synonymous mutations than non-synonymous mutations, are highlighted. Additional details on the number of mutations and sequence conservation are given in supplemental Fig. S1. Figure 2 and the figures following show the results using the standard DNA code. Results using the vertebrate mitochondrial DNA code are also included in figure S1, with the only major difference being that the codon in position 22 becomes a stop codon, yielding a 21 amino acid peptide instead of 24. A curious feature of the humanin sequences is that start codon methionine is not particularly well-conserved. Mutations of the start codon occur across all vertebrate branches with an overall conservation rate of 79%, ranging from 96% in birds to 46% in reptiles. The stop codon in position 25 is even less well conserved, with an overall conservation of 58%.

The results for vertebrates show two regions of highly conserved or invariant bases, corresponding to the codons for C8, L9 and E15, I16. This conservation might be due to these bases being critical for ribosome function, or it could be due to the corresponding amino acids being critical for humanin function, or a combination of both. On the other hand, there are highly conserved amino acids with high *f*_syn_ values in regions with less well conserved base sequences, A2, L10, D17, L18 and V20, suggesting that whereas these amino acids are important for humanin function, the corresponding bases are less important for ribosome function. The C-terminal region has highly conserved residues in mammals but shows more variation in non-mammals. Especially interesting is position 22, which in mammals is nearly always arginine, but in non-mammals is usually lysine, suggesting that a positive charge in this position is important for humanin function or processing. Some amino acids have high *f*_syn_ values in primates, F6 and R23, but not in other vertebrates, and vice versa, G5 and D17. This could reflect some specialization of function involving those residues present in one branch of vertebrates but absent in others, or the high *f*_syn_ values could be due to chance. The probability of a *f*_syn_ > 0.5 happening by chance is estimated empirically to be in the range 0.035–0.065, as described in a later section that examines the statistical significance of the MDP analyses (see Statistical analysis).

### MOTS-c contains a highly conserved pentapeptide sequence, MGYIF, but has almost no synonymous codon bias

**Figure 3:**
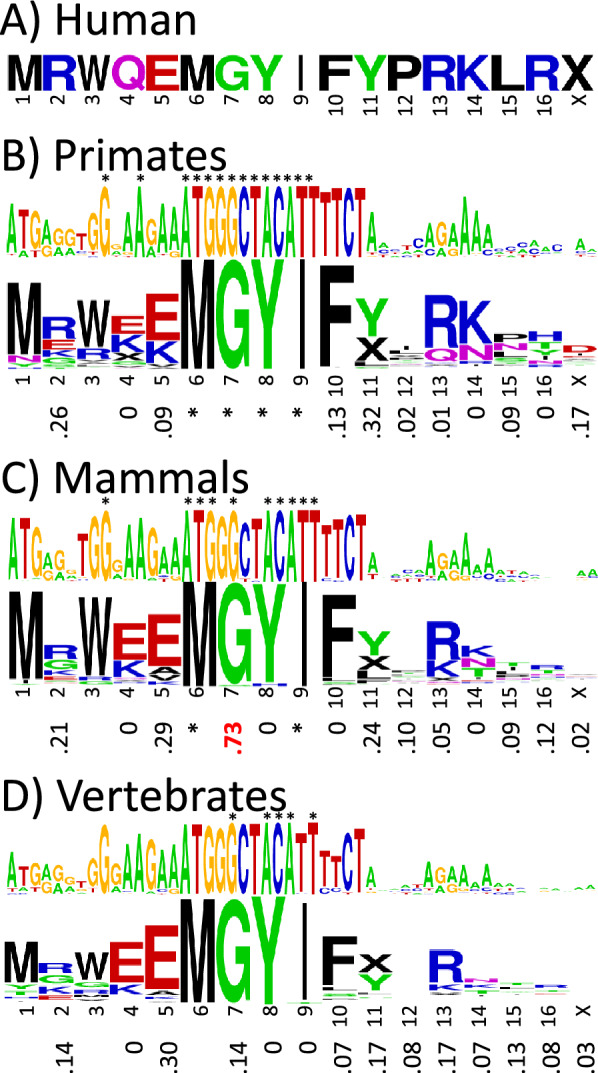
Synonymous codon bias in MOTS-c. (**A**) MOTS-c sequence in humans. Sequence logos of the base and amino acid sequence alignments with the synonymous codon bias *f*_syn_ below in (**B**) primates (number of species = 254), (**C**) mammals (number of species = 178), and (**D**) vertebrates (number of species = 348). The base and amino acid letter heights indicate how conserved they are across species. Invariant bases and amino acids with invariant codons are indicated with asterisks. The “X” symbol stands for stop codon. Values of *f*_syn_ ≥ 0.5 are highlighted in red. Because methionine and tryptophan have only one codon in the standard DNA code, they have no *f*_syn_ values. The color code for the amino acids is hydrophobic (black), acidic (red), basic (blue), and neutral hydrophilic (green for glycine and hydroxyl-containing residues and magenta for amide-containing).

Figure 3 shows the consensus base and amino acid sequence logos for the primate, mammal, and vertebrate MOTS-c alignments, with the corresponding *f*_syn_ values below. Additional details on the number of mutations, sequence conservation, and differences using the vertebrate mitochondrial DNA code are given in supplemental Fig. S2. The most striking feature of the MOTS-c alignments is the highly conserved pentapeptide MGYIF in the middle of the sequence. The N-terminal region shows only moderate sequence conservation, and the C-terminal region shows even less. In contrast to humanin, nearly all the *f*_syn_ values are small, apart from G7 in the alignment for mammals. Thus, full-length MOTS-c does not show compelling evidence for natural selection in vertebrates. However, given the very high conservation of the MGYIF pentapeptide with its high *f*_syn_ value for G7 in mammals, it is possible that the pentapeptide is biologically important, at least in mammals.

### SHLP1, SHLP3, & SHLP5 show poor start codon conservation and almost no synonymous codon bias

**Figure 4:**
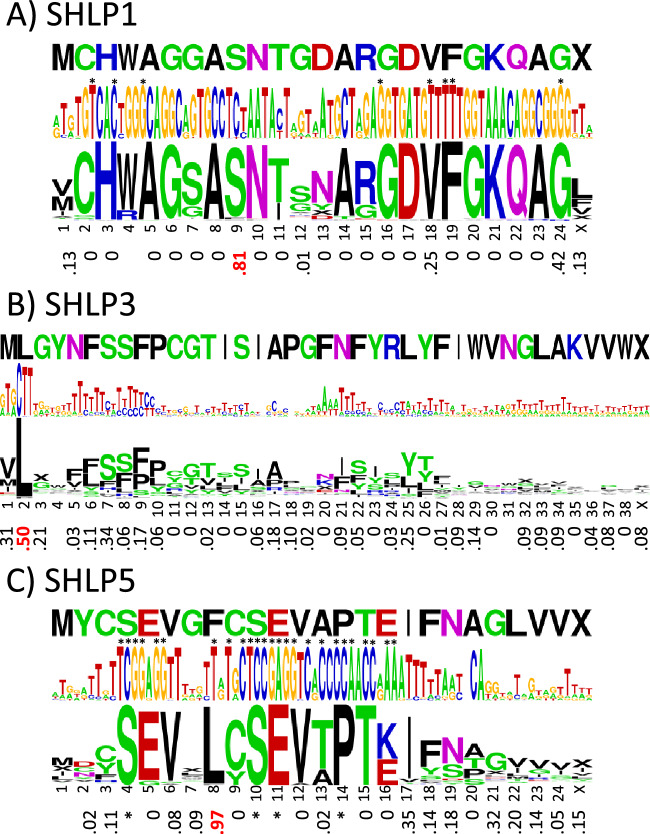
Synonymous codon bias in SHLP1, SHLP3, and SHLP5. (**A**) The human SHLP1 sequence above the corresponding sequence logos of the base and amino acid sequence alignments and synonymous codon bias *f*_syn_ values below in primates (number of species = 217). (**B**) The human SHLP3 sequence above the corresponding sequence logos of the base and amino acid sequence alignments and synonymous codon bias *f*_syn_ values below in primates (number of species = 221). (**C**) The human SHLP5 sequence above the corresponding sequence logos of the base and amino acid sequence alignments and synonymous codon bias *f*_syn_ values below in primates (number of species = 216). The base and amino acid letter heights indicate how conserved they are across species. Invariant bases and amino acids with invariant codons are indicated with asterisks. The “X” symbol stands for stop codon. Values of *f*_syn_ ≥ 0.5 are highlighted in red. Because methionine and tryptophan have only one codon in the standard DNA code, they have no *f*_syn_ values. The color code for the amino acids is hydrophobic (black), acidic (red), basic (blue), and neutral hydrophilic (green for glycine and hydroxyl-containing residues and magenta for amide-containing).

Figure 4 shows the consensus base and amino acid sequence logos for primates for the SHLP1, SHLP3 and SHLP5 peptides, with the corresponding *f*_syn_ values below. Additional details on the number of mutations, sequence conservation, and differences using the vertebrate mitochondrial DNA code are given in supplemental Fig. S3. None of these SHLP peptides show compelling evidence of natural selection in primates. The highlighted positions with high *f*_syn_ values, one per peptide, could easily be due to chance. In addition, their start codons are poorly conserved, 33% for SHLP1, 18% for SHLP3, and 30% for SHLP5, further arguing against important biological roles for these peptides. To confirm that SHLP1, SHLP3, and SHLP5 are not conserved in vertebrates, the human sequences were aligned with 19 non-primate vertebrate species (supplemental Table S37), again showing poor sequence conservation, especially with regard to the start codons. Because of this, full analyses with mammal and vertebrate multiple sequence alignments were not performed. Also, it should be noted that the poor conservation of the SHLP3 base sequence is somewhat misleading; it contains two regions with insertions/deletions of varying length, and when aligned allowing gaps, the base sequence conservation improves dramatically.

### SHLP2 shows poor sequence conservation and almost no synonymous codon bias but is preceded by a sequence with high synonymous codon bias, SHLP2b

**Figure 5:**
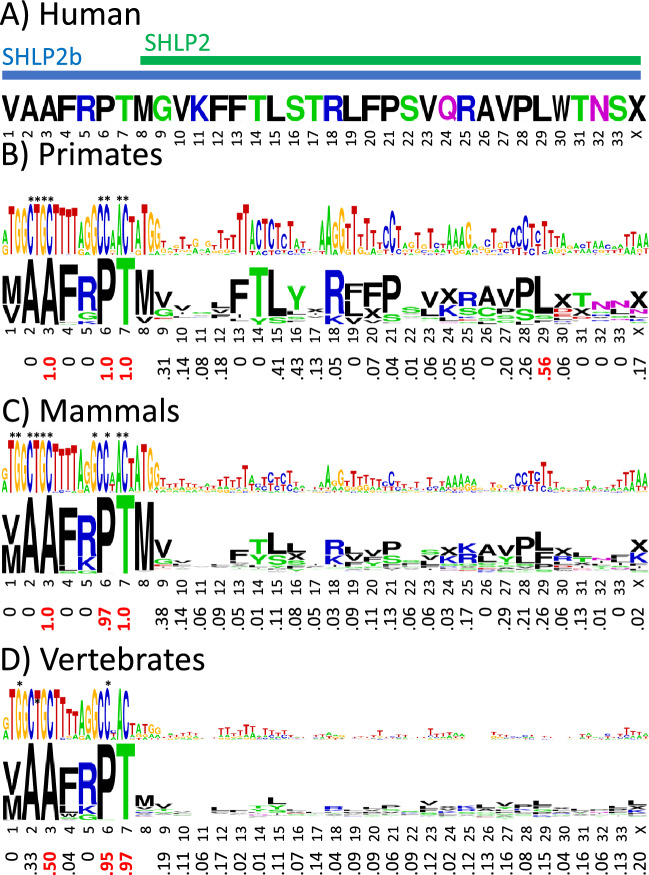
Synonymous codon bias in SHLP2 and SHLP2b. (**A**) SHLP2b (blue line) and SHLP2 (green line) sequences in humans. Sequence logos of the base and amino acid sequence alignments with the synonymous codon bias *f*_syn_ below in (**B**) primates (number of species = 219), (**C**) mammals (number of species = 174), and (**D**) vertebrates (number of species = 369). The base and amino acid letter heights indicate how conserved they are across species. Invariant bases are indicated with asterisks. The “X” symbol stands for stop codon. Values of *f*_syn_ ≥ 0.5 are highlighted in red. Because methionine has only one codon in the standard DNA code, it has no *f*_syn_ value. The color code for the amino acids is hydrophobic (black), acidic (red), basic (blue), and neutral hydrophilic (green for glycine and hydroxyl-containing residues and magenta for amide-containing).

Initial analysis of the SHLP2 sequence alignment revealed a cluster of codons with high *f*_syn_ values preceding SHLP2 in the same open reading frame. This cluster starts with a standard methionine ATG start codon in most primate species, and this new, putative peptide is named SHLP2b. Because they overlap, the consensus base and amino acid sequence logos for SHLP2 and SHLP2b are shown together in Fig. 5, with the corresponding *f*_syn_ values below. Additional details on the number of mutations, sequence conservation, and differences using the vertebrate mitochondrial DNA code are given in supplemental Fig. S4. Humans have the alternate start codon GTG at the start of the putative SHLP2b peptide, as do most non-primate vertebrates^18^. In humans, the stop codon for SHLP2b would be the same as for SHLP2, but for primates in general, the stop codon would more often be in position 24 in Fig. 5, yielding a peptide 23 residues long.

In primates SHLP2 has only one amino acid, in position 29 in Fig. 5, with an *f*_syn_ value greater than 0.5, but two others, in positions 15 and 16, have values close to 0.5. Because the choice of 0.5 as the *f*_syn_ threshold was somewhat arbitrary, SHLP2 was also analyzed at the mammal and vertebrate levels; however, the *f*_syn_ values were even lower. Thus, SHLP2 does not show compelling evidence of purifying selection. In contrast, the cluster of conserved, high *f*_syn_ value codons at the beginning of SHLP2b persists even at the vertebrate level, which suggests the putative SHLP2b peptide might be biologically important. Note that, like SHLP3, SHLP2 contains a region with insertion/deletions soon after the start codon, making the base sequence conservation appear misleadingly low in the figure.

### SHLP4 contains a well-conserved N-terminal region with residues showing synonymous codon bias

**Figure 6:**
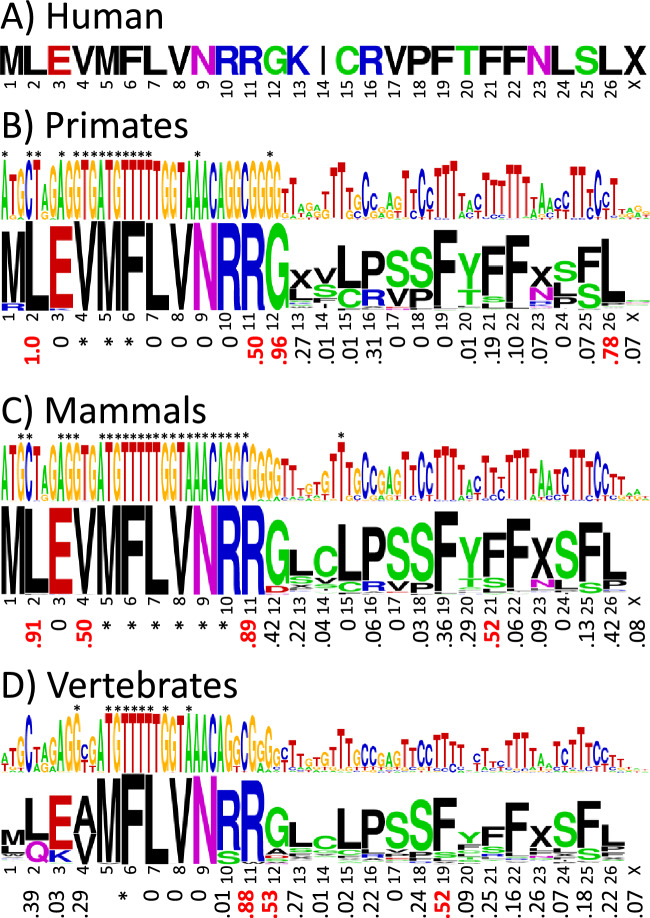
Synonymous codon bias in SHLP4. (**A**) SHLP4 sequence in humans. Sequence logos of the base and amino acid sequence alignments with the synonymous codon bias *f*_syn_ below in (**B**) primates (number of species = 215), (**C**) mammals (number of species = 144), and (**D**) vertebrates (number of species = 339). The base and amino acid letter heights indicate how conserved they are across species. Invariant bases and amino acids with invariant codons are indicated with asterisks. The “X” symbol stands for stop codon. Values of *f*_syn_ ≥ 0.5 are highlighted in red. Because methionine has only one codon in the standard DNA code, it has no *f*_syn_ value. The color code for the amino acids is hydrophobic (black), acidic (red), basic (blue), and neutral hydrophilic (green for glycine and hydroxyl-containing residues and magenta for amide-containing).

Figure 6 shows the consensus base and amino acid sequence logos for the primate, mammal, and vertebrate SHLP4 alignments, with the corresponding *f*_syn_ values below. Additional details on the number of mutations, sequence conservation, and differences using the vertebrate mitochondrial DNA code are given in supplemental Fig. S5. In mammals, including primates, the N-terminal region is highly conserved, with many invariant bases, and is flanked by amino acids, L2 and R11, for example, with high *f*_syn_ values. The results suggest that the N-terminal region of SHLP4, residues 1–12, might have undergone purifying selection in mammals. In contrast, the rest of the peptide is more poorly conserved. The alignments of SHLP4 have a region of insertions/deletions in the middle of the sequence, which accounts for the poorer sequence conservation. In non-mammal vertebrates, the start codon is not well-conserved, but M5 is still highly conserved, and nearly all mutations that do occur for it are to the alternate start codon GTG. The results including all vertebrates are not as compelling as for mammals alone, but it cannot be ruled out that residues 5–12 have also undergone natural selection in non-mammals, with M5 serving as the start codon.

### SHLP6 is the most conserved MDP with several residues showing synonymous codon bias

**Figure 7:**
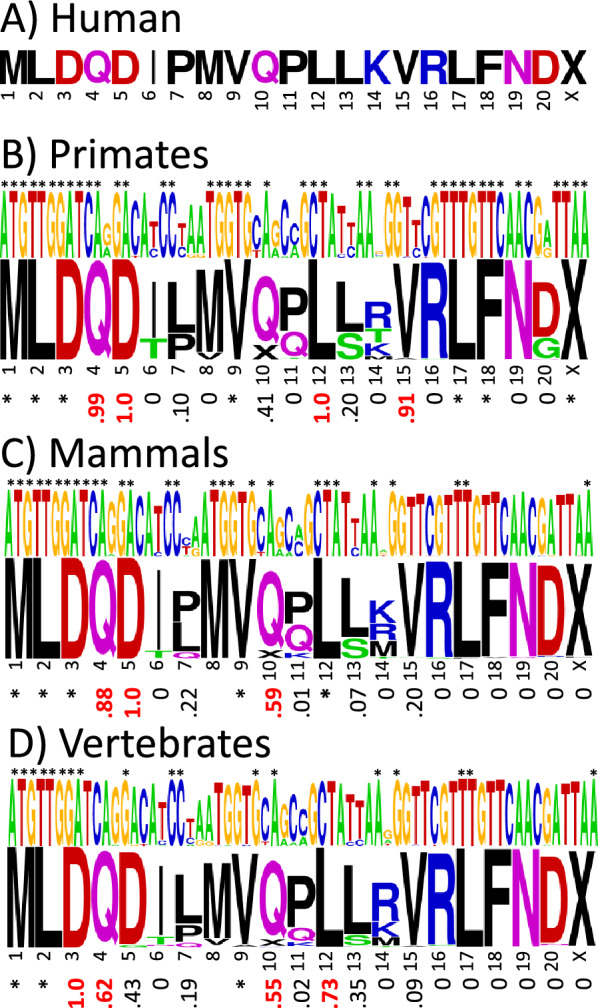
Synonymous codon bias in SHLP6. (**A**) SHLP6 sequence in humans. Sequence logos of the base and amino acid sequence alignments with the synonymous codon bias *f*_syn_ below in (**B**) primates (number of species = 242), (**C**) mammals (number of species = 147), and (**D**) vertebrates (number of species = 348). The base and amino acid letter heights indicate how conserved they are across species. Invariant bases and amino acids with invariant codons are indicated with asterisks. The “X” symbol stands for stop codon. Values of *f*_syn_ ≥ 0.5 are highlighted in red. Because methionine has only one codon in the standard DNA code, it has no *f*_syn_ value. The color code for the amino acids is hydrophobic (black), acidic (red), basic (blue), and neutral hydrophilic (green for glycine and hydroxyl-containing residues and magenta for amide-containing).

Figure 7 shows the consensus base and amino acid sequence logos for the primate, mammal, and vertebrate SHLP6 alignments, with the corresponding *f*_syn_ values below. Additional details on the number of mutations, sequence conservation, and differences using the vertebrate mitochondrial DNA code are given in supplemental Fig. S6. SHLP6 has the most highly conserved sequence of the MDPs presented here, and unlike the other MDPs, its stop codon is also highly conserved. In addition, it has several high *f*_syn_ value residues, thus the results suggest that the SHLP6 peptide has undergone purifying selection.

### Statistical analysis

As already mentioned, high *f*_syn_ values can happen by chance. The base sequences before and after the MDPs can be used to get an empirical estimate of the baseline probability that *f*_syn_ ≥ 0.5 occurs by chance because these regions lie outside the ORFs. In the sequence alignments, 12–15 additional bases were included before the start codon and after the stop codon, that is, 4–5 additional “codons” before and after each peptide. Using all the MDPs (for SHLP2/SHLP2b, only the bases before SHLP2b and after SHLP2 are used) this yields 77 additional “codons,” five of which have values *f*_syn_ ≥ 0.5, giving a probability estimate of 0.065. This empirical approach makes no assumptions about the neutrality of synonymous mutations except that they tend to occur more frequently in conserved peptide coding regions than non-coding regions. Because SHLP1, SHLP3 and SHLP5 show no evidence of natural selection, they can be used as a consistency check. Their sequence alignments each have just one amino acid position with *f*_syn_ ≥ 0.5. SHLP1 and SHLP5 are 24 amino acids long, and SHLP3 is 38. Assuming these high *f*_syn_ values are due to chance, this gives an average probability of 0.035, which suggests the probability of 0.065 calculated with the sequences before and after the MDPs could be an overestimate. The regions used for calculating the two baseline probability estimates consist of 231 bases before and after the MDPs ORFs, and 258 bases of SHLP1, 3 and 5, totaling 489 bases. The 16S gene length is 1558 bases; thus, a considerable amount of the RNA genes has been sampled to obtain the baseline probability estimates. Varying base conservation, presence of insertions/deletions, and proximity to ORFs could influence the baseline probability and might account for some of the difference in the two baseline values.

Table 1 shows the *p*-values for each sequence alignment using the binomial distribution (Eq. 2), using the more stringent baseline probability of 0.065. In other words, by using the higher estimate of the baseline probability, more residues of the MDP must have *f*_syn_ ≥ 0.5 to achieve statistical significance. Only humanin and SHLP6 have *p*-values less than 0.05, the usual criterion for considering results statistically robust, though SHLP4 is close. Keep in mind that because the value 0.065 could be an overestimate of the baseline probability, the *p*-values could also be overestimates.

**Table 1:** *p*-Values for the synonymous codon bias in MDP sequence alignments.

	Humanin	MOTS-c	SHLP1	SHLP2	SHLP2b^†^	SHLP3	SHLP4	SHLP5	SHLP6
Primates	**0.00063**	1.0	0.80	0.83	0.19	0.92	0.085	0.80	**0.037**
Mammals	**0.0037**	0.66	n/d	1.0	0.19	n/d	0.085	n/d	0.14
Vertebrates	**0.00063**	1.0	n/d	1.0	0.19	n/d	0.24	n/d	**0.037**

n/d not done.

^†^For SHLP2b, the peptide length in orangutans, 23, was used because orangutan is the species closest to humans that has an ATG start codon.

Significant values are given in bold.

## Discussion

Our evaluation of synonymous codon bias has revealed that at least two MDPs, humanin and SHLP6, show evidence of natural selection, implying these peptides have biological roles that have been important during the course of vertebrate evolution. For humanin the synonymous codon bias analysis in vertebrates shows robust evidence of purifying selection (p < 0.00063). Although the importance of humanin has already been demonstrated by knockdown experiments^5,17^, the result here serves as more than just a positive control. In humans there are 13 humanin homologs in the nuclear genome, 10 of which have been confirmed to be transcribed in various human tissues^19^. Nuclear DNA sequences originating from mitochondria are called numts (NUclear sequence of MiTochondrial origin), and they are common^20^. Thus, hypothetically, it could be that RNA interference with the nuclear versions, and not mitochondrial humanin, led to dysfunction in the knockdown experiments. Nevertheless, the result here suggests the mitochondrial version of humanin must be important because its peptide sequence has been conserved throughout vertebrate evolution. The nuclear versions might also be important, and they warrant further study, such as using CRISPR techniques to alter or knock out the numts, and methods of introducing base changes into mitochondrial genes are on the horizon^21^.

SHLP6 also shows synonymous codon bias (p < 0.037 in vertebrates) satisfying the significance threshold of p < 0.05. Perhaps the results for SHLP4 (p < 0.085 in mammals) and SHLP2b (p < 0.19 in vertebrates) also reflect purifying selection in their highly conserved N-terminal regions, but there are too few residues with high *f*_syn_ values to conclude this statistically. Similarly, this analysis cannot rule out that the highly conserved MGYIF pentapeptide in MOTS-c has undergone purifying selection to conserve its sequence, but the region is too short, with only G7 having a high *f*_syn_ value in mammals, to conclude significance. While this study did not include invertebrate species, searches of their mitochondrial DNA reveal sequences homologous to humanin, SHLP6 and the highly conserved regions of SHLP2b, SHLP4 and MOTS-c; so MDPs do not appear to be unique to vertebrates (supplemental Fig. S7).

For the other MDPs: SHLP1, SHLP2, SHLP3 and SHLP5, the analyses show poor sequence conservation and no evidence of synonymous codon bias. SHLP peptides were first identified by analyzing the human mitochondrial 16S gene *MT-RNR2* for all ORFs greater or equal to 20 amino acids long, and it is certainly possible that some of them arose by chance and do not correspond to biologically important peptides. The authors know of no reports showing biological activities for SHLP1 and SHLP5. In the original paper describing them, SHLP2 and SHLP3 were shown to be cytoprotective in two cell models, one of human and one of murine origin. Curiously, mice (*Mus musculus*) do not seem to have homologs of SHLP2 and SHLP3 (supplemental Fig. S8), thus, at least for the mouse cell model, the cytoprotective effect would seem serendipitous. It is also possible that SHLP1, SHLP2, SHLP3 and SHLP5 are biologically important, but they arose too recently in human evolution for synonymous codon analyses to be useful.

An earlier analysis of MOTS-c sequences in 14 species (13 mammals and 1 fish) claimed to show evidence of “positive” selection^15^. Positive selection typically refers to a recently introduced beneficial mutation that sweeps through a population, and it is unclear how this should be interpreted across species. Four residues were claimed to show positive selection, Q4, E5, G7 and I9, and the base sequences and the codon counts for these residues for the 14 species are reanalyzed here (supplemental Fig. S9). The bases for G7 are invariant, whereas those of Q4 are quite variable. The previously reported data showed ratios of non-synonymous to synonymous mutation rates, with larger ratios indicating positive selection, but because G7 is invariant, both rates are zero and it is unclear how the reported ratio was obtained. It is possible the results are artifacts due to the small sample size. In any case, none of the four residues has synonymous codons in the 14 species. Interestingly, G7 does have synonymous mutations in sloths (supplemental Table S10), but no sloths were included in the earlier analysis.

One feature that distinguishes humanin and SHLP6 from the other MDPs of the 16S gene *MT-RNR2* is the direction of their translation, with humanin and SHLP6 being coded by the H-strand of the mtDNA and the others being coded by the L-strand (Fig. 1). MOTS-c is also coded by the H-strand. Both strands of mtDNA are transcribed into RNA polycistronically over nearly their whole lengths. However, there are no genes coded by the L-strand region complementary to the 16S and 12S ribosomal genes, and transcription of the L-strand often terminates before reaching there^22^. This means that the L-strand coded MDPs would be less transcribed than the H-strand coded ones. MDP RNA sequences, including sequences with 3’ polyadenylated tails, have been observed in cell and plasma samples using humanin, MOTS-c and SHLP6 gene sequence probes in Northern blot and RT-PCR experiments^14,19,23,24^, but no such experiments have yet been reported for the other SHLP peptides.

SHLP6 is unique among the MDPs in that both its start and stop codons are nearly invariant. The relatively poor conservation of the start codon of humanin has been reported before^25^, and is confirmed here, with only 79% conservation over all vertebrate species. Even less conserved are the other MDP stop codons, with a 58% conservation rate for humanin, and only 17% in SHLP4, though SHLP4 has an earlier stop codon at position 23 in 71% of mammals. The arginine codon in position 22 of humanin is a stop codon in the vertebrate mitochondrial DNA code, and in mammals, it is nearly invariant with an *f*_syn_ value of 0.86 for the stop codon (supplemental Fig. S1). This suggests that purifying selection might be conserving the stop codon, but only if humanin is translated inside mitochondria. In fact, one study saw colocalization of the humanin peptide inside mitochondria in synovial cells from rheumatoid arthritis patients^26^, consistent with mitochondria being the site of humanin expression; however, more study is needed to determine whether this holds true under non-pathological conditions and in other cell types.

On the other hand, humanin has also been localized outside mitochondria, as well as extra-cellularly, and is known to act on both cytosolic and cell surface proteins^5–7,9,10,27^, thus, cytosolic translation is also a possibility. If humanin is translated in the cytosol, the resulting peptide can vary greatly in length; for instance, human humanin has 24 residues whereas rat humanin, also known as rattin, has 38. Intriguingly, humanin residues K21, R22, and R23, which are highly conserved in mammals, resemble the dibasic cleavage sites important in the processing of many secreted hormones^28^. SHLP4 residues R10 and R11, at the end of its highly conserved N-terminal region, also resemble a dibasic cleavage site, as do MOTS-c residues R13 and K14. Post-translational proteolytic processing could help explain why MDP stop codons are so variable; as long as the cleavage site is conserved, the sequence past it may be of little importance. However, it is not currently known whether proteolytic processing is important for MDP biological function. Also not known is whether MDPs are predominantly translated in mitochondria or in the cytosol. One argument for cytosolic translation is the fact that the second codon of MOTS-c is a stop codon in the mitochondrial DNA code, thus full-length MOTS-c must be translated in the cytosol^15^. However, the conserved pentapeptide MGYIF starting at position 6 of MOTS-c has its own start codon, so it could be translated inside mitochondria. The questions of proteolytic processing and mitochondrial versus cytosolic translation of MDPs clearly warrant further study.

One concern regarding the synonymous codon bias analysis is that out of the 55 residues with *f*_syn_ ≥ 0.5 in the MDP sequences, 15 are leucine residues. Leucine is one of three amino acids with six codons in the standard DNA code, and it is possible leucine is over-represented because it has a greater chance that a random mutation is synonymous. On the other hand, serine also has six codons, but only one of the residues with *f*_syn_ ≥ 0.5 is serine, so perhaps the effect is small. In fact, the reason for the higher number of leucine residues could be biological; the L9R and L10D mutants of humanin cannot be secreted from cells^29^, and perhaps leucine residues could be similarly important for other MDPs.

These shared features, variable peptide length, possible dibasic cleavage sites in humanin, SHLP4, and MOTS-c, and high leucine frequency in humanin and SHLP4 are consistent with these MDPs being processed and secreted, thereby implying roles in intercellular signaling. Indeed, both humanin and MOTS-c have already been detected outside cells^2,15,29^, though there are no reports yet on SHLP4. Conversely, the well-conserved stop codon of SHLP6 with a lack of any apparent dibasic cleavage site suggests its role might be intracellular. Intriguingly, if translated in mitochondria, humanin also has a highly conserved stop codon, replacing the dibasic cleavage site, suggesting humanin might play dual roles, intracellular for the peptide expressed in mitochondria and extracellular when expressed cytosolically.

The search for MDPs has focused on longer peptides, at least 20 residues in the case of the mitochondrial 16S gene, but the results presented here imply shorter peptides might also be important, especially if proteolytic processing occurs. In particular, the N-terminal regions of SHLP2b and SHLP4, and the MGYIF pentapeptide of MOTS-c are highly conserved and include residues with high *f*_syn_ values, suggesting they might have important biological roles. There are many biologically important shorter peptides, gonadotropin-releasing hormone with ten residues, oxytocin with nine, and the enkephalins with five, to name a few. MDPs need not occur only in RNA genes, and the synonymous codon bias analysis could be extended to identify candidate MDPs throughout the mitochondrial genome. One analysis suggested there could be over 80 ORFs leading to MDPs in the mitochondrial genome^30^, and an MDP outside the ribosomal genes, SHMOOSE, has already been identified^31^, as well as two microproteins, gau and Mtaltnd4^3,4^. In addition, by analyzing larger regions of the mitochondrial genome, including more non-peptide-coding base sequences, the statistical analysis can be improved.

The peptides belonging to the family of MDPs are fascinating, and they offer a new paradigm to regulate cell signaling, metabolism, inflammation, and cell death from within the mitochondrial genome. Some of them, such as humanin and MOTS-c have been studied more extensively than the others for their impact on the above cellular functions. Because of their reported beneficial effects, humanin and MOTS-c are already being marketed as anti-aging therapeutics and performance enhancers in humans^32^, in spite of the possible cancer promoting properties of humanin. This highlights the need to evaluate the potential validity of these peptides at the genome level. One outcome from such an evaluation is to ensure that the form of the peptide targeted for therapeutics is the relevant one. For instance, the results presented here suggest that the MOTS-c peptide as it is currently being studied (and marketed) might not be in its most biologically relevant form. Plus, identifying the important and conserved residues will aid optimization of the therapeutic performance of these peptides. At the fast rate of which these peptides are being developed for potential treatments of diseases against the backdrop of their potentially negative effects, it is only prudent to urge that they be subjected to the rigorous scrutiny of the broader scientific community.

## Methods

### Multiple sequence alignments

Sequence alignments of the base sequences coding the MDPs were assembled using nucleotide BLAST. Because the sequences correspond to open reading frames, the alignments contain no insertions or deletions. For the primate alignments, comprehensive, though not exhaustive, searches of all primate species were performed. For the vertebrate alignments, the sequences were limited to species whose nuclear genomes have also been sequenced, which yielded numbers of species comparable to the primate alignments. The resulting vertebrate alignments consisted of roughly 10% primates, 30% other mammals, 20% other land vertebrates, and 40% fish. For the mammal alignments, non-mammal sequences were deleted from the vertebrate list. For species with more than one version of the sequence, presumably due to polymorphisms, the most common version was used. For species that had a mutation of an otherwise invariant base, or a frame shifting insertion/deletion that resulted in mutation of otherwise invariant amino acids, the search was expanded to include any additional species with mutations at that site. If no other species with a mutation at that site were found, then the sequence was removed. As a consequence, the number of species in the alignments is variable, and the specific numbers are given in the figure legends. All the sequence alignments also included 12–15 bases before the start codon and 12–15 bases after the stop codon to provide a comparison with adjacent, presumably non-peptide-coding regions. All sequence alignments are included in the supplemental information (supplemental Tables S1–S36).

### Synonymous codon bias

To calculate the fraction of synonymous mutations, *f*_syn_, for each amino acid position, the most frequent codon of the consensus amino acid was taken as the “ancestral” codon, and the total number of synonymous mutations of that codon, *n*_syn_, is divided by the total number of mutations, synonymous plus non-synonymous, *n*_non_, at that position,

1 $${f}_{syn}= {n}_{syn}/\left({n}_{syn}+{n}_{non}\right)$$

Thus, a result of 0.0 indicates all mutations are non-synonymous, 0.5 indicates an equal number of synonymous and non-synonymous mutations, and 1.0 indicates all the mutations are synonymous for that position. For comparison, the ratio of synonymous to non-synonymous rates, which is another common way to express synonymous codon bias^1^, ranges from 0 for all non-synonymous mutations, 1 for an equal number of synonymous and non-synonymous mutations, to +$\infty$ for all synonymous mutations.

The one-tailed *p*-values for the number of occurrences of *f*_syn_ ≥ 0.5 in each sequence alignment were calculated using the binomial distribution,

2 $$p{\text{-value }} =\sum_{x=X}^{n}\left(\frac{n!}{x!\left(n-x\right)!}\right){p}^{x}{(1-p)}^{(n-x)}$$

where *X* is the number of residues with *f*_syn_ ≥ 0.5, *n* is the length of the peptide and *p* is the random probability of *f*_syn_ ≥ 0.5. The probability p was calculated using non-peptide-coding regions of the vertebrate sequence alignments, except for SHLP1, SHLP3, and SHLP5, where the primate sequence alignments were used. The consensus sequence logo figures were made with Weblogo^33^.

### Supplementary Information

Supplementary Figures.

Supplementary Tables.

## Data Availability

All data generated or analyzed during this study are included in this published article and its supplementary information files.
