# Supplementary material for: Evidence of natural selection in the mitochondrial-derived peptides humanin and SHLP6
Source: Sci Rep. 2023 Aug 29;13:14110. doi: 10.1038/s41598-023-41053-0 (PMC10465549; doi:10.1038/s41598-023-41053-0)
Supplement: Supplementary file 1 — Supplementary Figures. [file 41598_2023_41053_MOESM1_ESM.pdf]

Evidence of natural selection in the mitochondrial-derived peptides humanin and SHLP6

James M. Gruschus*, Daniel L. Morris, Nico Tjandra

Supplemental Files

Figure S1. Synonymous (syn), non-synonymous (non) mutation counts for humanin, fraction conserved (cons) and fraction of synonymous mutations (fsyn), with vertebrate mitochondrial code synonymous (mtsyn) and non-synonymous (mtnon) counts.

Primates
nspec 252
>consensus

	x	G	L	V	w	M	A	T	R	G	F	N	C	L	L	L	L	I	S	E	I	D	L	S	V	K	r	R	I	x	N	N	K	T	r
	TAG	GGA	CTT	GTA	TGA	ATG	GCC	ACA	CGA	GGG	TTT	AAC	TGT	CTC	TTA	CTT	TTA	ATC	AGT	GAA	ATT	GAC	CTG	TCC	GTG	AAG	AGG	CGG	ATA	TAA	AAT	AAT	AAG	ACG	AGA
syn	112	2	0	0	0	n/a	133	0	0	1	66	0	0	6	18	8	13	0	0	0	0	2	154	3	0	4	38	21	0	38	32	21	0	1	0
non	24	1		145	1	72	2	86	1	2	46	38	0	0	7	12	131	106	0	0	1	8	11	127	4	5	0	2	188	119	177	7	1	1	1
cons	.90	.99	.99	.42	.99	.71	.99	.66	.99	.99	.82	.85	1.0	1.0	.97	.95	.48	.58	1.0	1.0	.99	.97	.96	.50	.98	.98	1.0	.99	.25	.53	.29	.97	.99	.99	.99
fsyn	.82	.67	.97	0	0	n/a	.99	0	0	.33	.54	0	n/a	1.0	.72	.40	.09	0	n/a	n/a	0	.20	.93	.02	0	.44	1.0	.91	0	.24	.15	.75	0	.50	0
mtsyn	99				0	0			0									0			0						38	21	0	8					0
mtnon	34				1	72			1									106			1						0	2	188	149					1

Mammals
nspec 148
>consensus

	x	G	L	V	w	M	A	T	R	G	F	N	C	L	L	L	P	I	S	E	I	D	L	P	V	K	r	R	E	x	N	N	K	T	r
	TAG	GGA	CTT	GTA	TGA	ATG	GCC	ACA	CGA	GGG	TTT	AAC	TGT	CTC	TTA	CTT	CCA	ATC	AGT	GAA	ATT	GAC	CTT	CCC	GTG	AAG	AGG	CGG	GAA	TAA	AAC	AAT	AAG	ACG	AGA
syn	61	1	46	0	0	n/a	65	0	0	9	31	0	0	2	12	12	4	0	0	0	0	1	63	7	0	1	6	6	0	14	18	18	0	0	0
non	18	5	4	34	1	38	1	66	1	11	18	73	0	0	9	26	80	28	14	1	1	4	10	25	1	11	1	11	74	58	110	20	1	0	0
cons	.88	.97	.97	.77	.99	.74	.99	.55	.99	.93	.88	.51	1.0	1.0	.94	.82	.46	.81	.91	.99	.99	.97	.93	.83	.99	.93	.95	.93	.50	.61	.26	.86	.99	1.0	1.0
fsyn	.77	.17	.92	0	0	n/a	.98	0	0	.45	.63	0	n/a	1.0	.57	.32	.05	0	0	0	0	.20	.86	.22	0	.08	.86	.35	0	.19	.14	.47	0	n/a	n/a
mtsyn	57				0	0			0									0			0						6	6		6					0
mtnon	22				1	38			1									28			1						1	11		66					0

Vertebrates
nspec 359
>consensus

	x	r	L	V	w	M	A	K	R	G	L	N	C	L	L	L	P	I	S	E	I	D	L	P	V	Q	K	R	E	x	T	H	K	T	r
	TAG	AGA	CTT	GTA	TGA	ATG	GCA	AAA	CGA	GGG	CTT	AAC	TGT	CTC	TTA	CTT	CCA	ATC	AGT	GAA	ATT	GAT	CTC	CCC	GTG	CAG	AAG	CGG	GAA	TAA	ACC	CAT	AAG	ACG	AGA
syn	169	4	85	0	0	n/a	214	0	0	72	24	0	0	2	141	21	22	1	1	0	0	153	203	51	28	77	11	25	0	30	29	6	0	8	0
non	100	180	141	53	4	74	21	251	14	43	197	125	0	0	60	220	225	147	164	20	32	24	35	68	5	153	163	82	233	150	300	198	13	4	16
cons	.72	.50	.61	.85	.99	.79	.94	.30	.96	.88	.45	.65	1.0	1.0	.83	.39	.37	.59	.54	.94	.91	.93	.90	.81	.99	.57	.55	.77	.35	.58	.16	.45	.96	.99	.96
fsyn	.63	.02	.38	0	0	n/a	.91	0	0	.63	.11	0	n/a	1.0	.70	.09	.09	.01	.01	0	0	.86	.85	.43	.85	.33	.06	.23	0	.17	.09	.03	0	.67	0
mtsyn	82	4				0			0									1			0							25		10					0
mtnon	187	180				74			14									147			32							82		170					16

Figure S2. Synonymous (syn), non-synonymous (non) mutation counts for MOTS-c, fraction conserved (cons) and fraction of synonymous mutations (fsyn), with vertebrate mitochondrial code synonymous (mtsyn) and non-synonymous (mtnon) counts.

Primates
nspec 254
>consensus

	V	K	V	x	P	M	r	W	E	E	M	G	Y	I	F	Y	I	r	K	P	H	D	T	L	Y	E
	GTC	AAG	GTG	TAG	CCC	ATG	AGA	TGG	GAA	GAA	ATG	GGC	TAC	ATT	TTC	TAC	ATC	AGA	AAA	CCC	CAC	GAT	ACT	CTT	TAT	GAA
syn	0	0	0	11	77	n/a	52	n/a	0	10	n/a	0	0	0	1	55	5	1	0	19	0	35	32	26	0	0
non	43	43	43	87	129	43	145	80	166	96	0	0	0	0	7	117	203	74	93	187	184	172	198	182	151	149
cons	.83	.83	.83	.66	.49	.83	.43	.69	.35	.62	1.0	1.0	1.0	1.0	.97	.54	.20	.71	.63	.26	.28	.32	.22	.28	.41	.41
fsyn	0	0	0	.11	.37	n/a	.26	n/a	0	.09	n/a	n/a	n/a	n/a	.13	.32	.02	.01	0	.09	0	.17	.14	.13	0	0
mtsyn				11		0	52	0			0			0			4	7								
mtnon				87		43	145	80			0			0			204	68								

Mammals
nspec 178
>consensus

	V	K	V	x	P	M	r	W	E	E	M	G	Y	I	F	Y	P	r	K	T	R	x	P	L	w	N
	GTC	AAG	GTG	TAG	CCT	ATG	AGG	TGG	GAA	GAA	ATG	GGC	TAC	ATT	TTC	TAC	CCC	AGA	AAA	ACC	AGG	TAA	CCT	TTA	TGA	AAC
syn	0	0	0	47	85	n/a	30	n/a	0	20	n/a	11	0	0	0	32	16	4	0	14	18	3	14	18	0	11
non	6	6	6	13	38	14	115	21	74	50	0	4	7	0	18	99	152	72	126	145	136	156	136	138	137	137
cons	.97	.97	.97	.93	.79	.92	.35	.88	.58	.72	1.0	.98	.96	1.0	.90	.44	.15	.60	.29	.19	.24	.12	.24	.22	.23	.23
fsyn	0	0	0	.78	.69	n/a	.21	n/a	0	.29	n/a	.73	0	0	0	.24	.10	.05	0	.09	.12	.02	.09	.12	0	.07
mtsyn						8	31	0			0			0				14							0	
mtnon						6	114	21			0			0				62							137	

Vertebrates
nspec 348
>consensus

	V	K	V	x	P	M	r	W	E	E	M	G	Y	I	F	x	T	r	N	T	R	K	K	L	w	K
	GTC	AAG	GTG	TAG	CCT	ATG	AGA	TGG	GAA	GAA	ATG	GGC	TAC	ATT	TTC	TAA	ACT	AGA	AAC	AAC	CGA	AAA	AAA	CTG	TGA	AAA
syn	0	0	1	54	67	n/a	40	n/a	0	29	n/a	1	0	0	7	42	25	28	21	43	24	9	19	35	0	3
non	87	187	67	84	229	120	248	159	121	69	5	6	1	15	97	204	304	138	274	284	260	300	307	294	270	262
cons	.75	.46	.81	.76	.34	.66	.29	.54	.65	.80	.99	.98	.99	.96	.72	.41	.13	.60	.21	.18	.25	.14	.12	.16	.22	.25
fsyn	0	0	.01	.39	.23	n/a	.14	n/a	0	.30	n/a	.14	0	0	.07	.17	.08	.17	.07	.13	.08	.03	.06	.11	0	.01
mtsyn				54		14	40	0			0			0		30		26			24				2	
mtnon				84		107	248	159			6			15		216		140			260				268	

Figure S3. Synonymous (syn), non-synonymous (non) mutation counts for primate SHLP1, SHLP3, SHLP5, fraction conserved (cons) and fraction of synonymous mutations (fsyn), with vertebrate mitochondrial code synonymous (mtsyn) and non-synonymous (mtnon) counts.

SHLP1 Primates
nspec 217
> consensus

	P	R	P	L	N	V	C	H	W	A	G	S	A	S	N	T	S	N	A	r	G	D	V	F	G	K	Q	A	G	L	S	F	A	E
	CCG	CGG	CCG	TTA	AAC	GTG	TGT	CAC	TGG	GCA	GGC	AGT	GCC	TCT	AAT	ACT	AGT	AAT	GCT	AGA	GGT	GAT	GTT	TTT	GGT	AAA	CAG	GCG	GGG	TTA	AGT	TTT	GCC	GAG
syn	0	2	0	72	4	19	0	0	n/a	0	0	0	0	42	0	0	1	0	0	0	0	0	1	0	0	0	0	0	3	17	1	0	3	6
non	5	3	5	19	5	130	17	1	34	1	4	54	4	10	6	61	114	77	13	58	3	3	3	3	7	6	6	6	4	115	171	76	78	112
cons	.98	.99	.98	.91	.98	.40	.92	.99	.84	.99	.98	.75	.98	.95	.97	.72	.47	.65	.94	.73	.99	.99	.99	.99	.97	.97	.97	.97	.98	.47	.21	.65	.64	.48
fysn	0	.40	0	.79	.44	.13	0	0	n/a	0	0	0	0	.81	0	0	.01	0	0	0	0	0	.25	0	0	0	0	0	.42	.13	.01	0	.04	.05
mtsyn		2							0											0														
mtnon		3							34											58														

SHLP3 Primates
nspec 221
>consensus

	S	L	C	F	F	V	L	x	W	F	F	S	S	F	P	C	G	T	S	S	i	A	P	G
	AGT	CTT	TGC	TTT	TTT	GTG	CTT	TAA	TGG	TTT	TTT	TCA	TCT	TTC	CCT	TGC	GGT	ACT	TCT	TCT	ATA	GCG	CCA	GGT
syn	3	0	0	1	4	40	6	42	n/a	4	18	45	8	23	8	0	0	3	0	0	9	24	20	3
non	135	100	2	103	54	90	6	155	169	129	141	89	128	109	125	174	132	129	177	136	143	112	172	186
cons	.39	.55	.99	.53	.76	.59	.97	.30	.24	.42	.36	.60	.42	.51	.43	.21	.40	.42	.20	.38	.35	.49	.22	.16
fsyn	.02	0	0	.01	.07	.31	.50	.21	n/a	.03	.11	.34	.06	.17	.06	0	0	.02	0	0	.06	.18	.10	.02
mtsyn								25	4												8			
mtnon								172	165												144			

	N	I	S	I	S	Y	T	F	I	G	N	W	w	V	V	F	L	G	F	x	V	L	L	V
	AAT	ATT	TCT	ATC	TCC	TAT	ACT	TTT	ATT	GGT	AAT	TGG	TGA	GTG	GTT	TTT	TTG	GGG	TTT	TAG	GTG	TTG	TTG	GTG
syn	0	13	7	0	6	37	0	2	17	28	0	n/a	17	19	17	0	7	15	0	15	21	10	20	14
non	168	126	139	146	166	109	146	177	178	172	188	179	174	186	181	193	188	183	183	179	185	183	172	182
cons	.24	.43	.37	.34	.25	.51	.34	.20	.19	.22	.15	.19	.21	.16	.18	.13	.15	.17	.17	.19	.16	.17	.22	.18
fsyn	0	.09	.05	0	.03	.25	0	.01	.09	.14	0	n/a	.09	.09	.09	0	.04	.08	0	.08	.10	.05	.10	.07
mtsyn		0		0					0			6	15							10				
mtnon		139		146					199			173	176				184							

SHLP5 Primates
nspec 216
>consensus

	L	C	G	L	S	M	D	C	S	E	V	x	L	C	S	E	V	T	P	T	K	I	F	N	A	G	V	V	V	x	C	L	L	G
	TTG	TGT	GGT	CTT	AGC	ATG	GAT	TGT	TCG	GAG	GTT	TAG	TTA	TGC	TCC	GAG	GTC	ACC	CCA	ACC	AAA	ATT	TTT	AAT	GCA	GGT	GTA	GTA	GTT	TAG	TGC	CTG	TTG	GGT
syn	32	4	6	60	26	n/a	4	16	0	0	1	17	114	0	0	0	0	1	0	0	0	12	14	21	0	46	35	23	8	26	7	9	30	19
non	105	155	124	39	125	151	162	124	0	15	12	162	4	47	0	0	7	54	0	1	92	22	88	97	143	96	139	137	145	147	183	158	139	148
cons	.51	.28	.43	.82	.42	.30	.25	.43	1.0	.93	.94	.25	.98	.78	1.0	1.0	.97	.75	1.0	.99	.57	.90	.59	.55	.34	.56	.36	.37	.33	.32	.15	.27	.36	.31
fsyn	.22	.03	.05	.34	.17	n/a	.02	.11	n/a	0	.08	.09	.97	0	n/a	n/a	0	.02	n/a	0	0	.35	.14	.18	0	.32	.20	.14	.05	.15	.04	.05	.18	.11
mtsyn						30						11										12								25				
mtnon						121						168										22								148				

Figure S4. Synonymous (syn), non-synonymous (non) mutation counts for SHLP2b and SHLP2, fraction conserved (cons) and fraction of synonymous mutations (fsyn), with vertebrate mitochondrial code synonymous (mtsyn) and non-synonymous (mtnon) counts.

Primates
nspec 219
>Consensus

	T	L	S	x	L	M	A	A	F	r	P	T	M	V	V	G	L	F	T	L	Y	x	r	F	F	P	S	V	x	r	A	V	P	L	x	T	N	N	x	A	Y	r	G
	ACG	CTT	TCT	TAA	TTG	ATG	GCT	GCT	TTT	AGG	CCA	ACT	ATG	GTA	GTT	GGG	TTG	TTT	ACT	CTC	TAT	TAA	AGG	TTT	TTT	CCT	AGT	GTC	TAA	AGA	GCT	GTC	CCT	CTT	TAG	ACT	AAC	AAT	TAA	GCT	TAC	AGG	GGG
syn	0	0	0	16	3	n/a	0	1	0	0	43	16	n/a	48	23	15	33	0	0	24	60	22	2	0	7	2	1	7	7	6	0	26	27	60	7	0	0	0	22	1	3	12	22
non	0	8	0	15	9	92	4	0	14	60	1	0	37	108	144	166	151	54	30	35	81	157	42	128	90	52	136	107	121	116	103	103	77	47	116	115	132	146	104	172	103	149	121
cons	1.0	.96	1.0	.93	.95	.58	.98	1.0	.94	.73	.99	1.0	.83	.51	.34	.24	.31	.75	.86	.84	.63	.30	.81	.42	.59	.76	.38	.51	.45	.47	.53	.53	.65	.79	.47	.47	.40	.33	.53	.21	.53	.32	.45
fsyn	0	0	0	.52	.25	n/a	0	1.0	0	0	.98	1.0	n/a	.31	.14	.08	.18	0	0	.41	.43	.13	.05	0	.07	.04	.01	.06	.05	.05	0	.20	.26	.56	.06	0	0	0	.17	.01	.03	.07	.15
mtsyn				3		2				0			3									5	2						2	5					16				2			15	
mtnon				28		90				60			34									174	42						126	117					107				124			146	

Mammals
nspec 174
>Consensus

	T	L	S	x	L	V	A	A	F	r	P	T	M	V	V	V	L	F	T	L	L	i	r	L	V	P	S	S	x	K	A	V	P	L	x	L	N	F	x	I	Y	L	G
	ACG	CTT	TCT	TAA	TTG	GTG	GCT	GCT	TTT	AGG	CCA	ACT	ATG	GTT	GTT	GTT	TTA	TTT	ACT	CTC	CTA	ATA	AGG	TTG	GTA	CCT	AGT	CTA	TAA	AAG	GCT	GTC	CCT	CTT	TAG	CTA	AAC	TTT	TAA	ATT	TAC	TTA	GGG
syn	0	2	0	2	0	0	0	3	0	0	70	26	n/a	48	21	9	14	5	1	10	10	8	3	13	14	15	9	9	4	23	0	42	22	32	7	19	2	0	2	7	4	11	10
non	0	2	5	44	4	87	2	0	23	53	2	0	10	79	134	147	145	94	97	83	120	147	95	125	124	99	139	147	116	115	106	105	85	93	116	129	141	134	108	131	114	139	148
cons	1.0	.99	.97	.75	.98	.50	.99	.98	.87	.70	.99	1.0	.94	.55	.23	.16	.17	.46	.44	.52	.31	.16	.45	.28	.29	.43	.20	.16	.33	.34	.39	.40	.51	.47	.33	.26	.19	.23	.38	.25	.34	.20	.15
fsyn	0	.50	0	.04	0	0	0	1.0	0	0	.97	1.0	n/a	.38	.14	.06	.09	.05	.01	.11	.08	.05	.03	.09	.11	.13	.06	.06	.03	.17	0	.29	.21	.26	.06	.13	.01	0	.02	.05	.03	.07	.06
mtsyn				2						0			1									4	2						10						40				2	0			
mtnon				44						53			9									151	96						110						83				108	138			

Vertebrates
nspec 369
>consensus

	T	L	S	x	L	V	A	A	F	r	P	T	M	V	V	V	L	F	T	L	L	L	r	L	L	P	S	V	x	K	L	V	P	L	x	L	P	L	L	I	Y	L	L
	ACG	CTT	TCT	TAA	TTG	GTG	GCT	GCT	TTT	AGG	CCC	ACT	ATG	GTT	GTT	GTT	CTT	TTT	TAT	CTC	CTA	CTT	AAG	TTG	TTG	TCT	AGT	TTT	TAA	AAG	CTG	GTT	CCT	CTT	TAG	TTA	CCC	TTT	TTG	ATT	TAC	TTA	CTT
syn	0	12	3	6	1	0	2	3	5	0	134	111	n/a	59	37	22	53	5	2	30	21	47	12	29	32	19	32	39	7	38	42	60	23	47	12	55	19	46	67	20	4	45	42
non	38	83	71	228	142	161	4	3	108	100	7	4	204	254	309	322	255	282	290	254	302	288	275	299	309	277	310	280	301	285	276	304	275	271	305	284	305	296	272	305	307	299	310
cons	.90	.78	.81	.38	.62	.56	.99	.99	.71	.73	.98	.99	.45	.31	.16	.13	.31	.24	.21	.31	.18	.22	.25	.19	.16	.25	.16	.24	.18	.23	.25	.18	.25	.27	.17	.23	.17	.20	.26	.17	.17	.19	.16
fsyn	0	.13	.04	.03	.01	0	.33	.50	.04	0	.95	.97	n/a	.19	.11	.06	.17	.02	.01	.11	.07	.14	.04	.09	.09	.06	.09	.12	.02	.12	.13	.16	.08	.15	.04	.16	.06	.13	.20	.06	.01	.13	.12
mtsyn				3						0			7										34						14						43					0			
mtnon				231						100			197										251						294						274					325			

Figure S5. Synonymous (syn), non-synonymous (non) mutation counts for SHLP4, fraction conserved (cons) and fraction of synonymous mutations (fsyn), with vertebrate mitochondrial code synonymous (mtsyn) and non-synonymous (mtnon) counts.

Primates
nspec 215
>consensus

	P	L	i	L	V	M	L	E	V	M	F	L	V	N	r	R	G	x	V	L	P	S	S	F	Y	F	F	x	S	F	L	G	A	C	L	C
	CCT	CTA	ATA	CTA	GTA	ATG	CTA	GAG	GTG	ATG	TTT	TTG	GTA	AAC	AGG	CGG	GGT	TAG	GTT	TTG	CCG	AGT	TCC	TTT	TAC	TTT	TTT	TAA	TCT	TTC	CTT	GGG	GCA	TGA	CTG	TGT
syn	0	1	0	101	31	n/a	53	0	0	0	0	0	0	0	0	2	48	42	1	1	35	0	0	0	1	12	3	8	0	7	92	22	6	0	10	0
non	1	51	2	19	59	23	0	7	0	0	0	2	3	2	2	2	2	112	134	75	79	82	63	14	81	50	26	109	125	96	26	173	73	123	76	74
cons	.99	.76	.99	.91	.73	.89	1.0	.97	1.0	1.0	1.0	.99	.99	.99	.99	.99	.99	.48	.38	.65	.63	.62	.71	.93	.62	.77	.88	.49	.42	.55	.88	.20	.66	.43	.65	.66
fsyn	0	.01	0	.84	.34	n/a	1.0	0	*	*	*	0	0	0	0	.50	.96	.27	.01	.01	.31	0	0	0	.01	.19	.10	.07	0	.07	.78	.11	.08	0	.12	0
mtsyn			0			0									0			40										16								
mtnon			2			23									2			114										101								

Mammals
nspec 144
>consensus

	P	L	i	L	V	M	L	E	V	M	F	L	V	N	r	R	G	L	C	L	P	S	S	F	Y	F	F	x	S	F	L	x	A	C	L	C
	CCT	CTA	ATA	CTA	GTA	ATG	CTA	GAG	GTA	ATG	TTT	TTG	GTA	AAC	AGG	CGG	GGT	TTG	TGT	TTG	CCG	AGT	TCC	TTT	TAC	TTC	TTT	TAA	TCT	TTC	CTT	TAA	GCA	TGC	CTG	TGT
syn	0	3	0	77	33	n/a	29	0	2	n/a	0	0	0	0	0	8	14	17	2	0	2	0	1	4	17	45	1	4	0	4	26	11	1	0	10	0
non	1	31	2	12	60	3	3	2	2	0	0	0	0	0	0	1	19	62	54	59	31	31	28	7	42	42	15	41	36	27	36	122	72	119	73	65
cons	.99	.78	.99	.92	.58	.98	.98	.99	.99	1.0	1.0	1.0	1.0	1.0	1.0	.99	.87	.57	.63	.59	.78	.78	.81	.95	.71	.71	.90	.71	.75	.81	.75	.15	.50	.17	.49	.55
fsyn	0	.09	0	.87	.35	n/a	.91	0	.50	n/a	n/a	n/a	n/a	n/a	n/a	.89	.42	.22	.04	0	.06	0	.03	.36	.29	.52	.06	.09	0	.13	.42	.08	.01	0	.12	0
mtsyn			0			0				0					0	8												3				18				
mtnon			2			3				0					0	1												42				115				

Vertebrates
nspec 339
>consensus

	L	i	L	V	M	L	E	A	M	F	L	V	N	r	R	G	L	C	L	P	S	S	F	Y	F	F	x	S	F	L	x	A	T	L	C
	CTA	ATA	CTT	GTA	ATG	CTA	GAG	GCG	ATG	TTT	TTG	GTA	AAC	AGG	CGG	GGT	TTG	TGT	TTG	CCG	AGT	TCC	TTT	TAC	TTT	TTT	TAA	TCT	TTC	CTT	TAA	GCA	ACT	CTG	TGT
syn	69	8	125	65	n/a	81	2	63	n/a	0	0	0	0	1	111	115	51	2	2	28	0	24	77	21	57	14	53	8	20	44	21	30	21	21	0
non	153	134	152	220	190	125	73	156	10	0	1	7	3	66	15	102	139	177	95	100	103	74	70	219	169	73	149	111	92	160	294	228	285	211	224
cons	.55	.60	.55	.35	.44	.63	.78	.54	.97	1.0	.99	.98	.99	.81	.96	.70	.59	.48	.72	.71	.70	.78	.79	.35	.50	.78	.56	.67	.75	.53	.13	.33	.16	.38	.34
fsyn	.31	.06	.45	.23	n/a	.39	.03	.29	n/a	n/a	0	0	0	.01	.88	.53	.27	.01	.02	.22	0	.24	.52	.09	.25	.16	.26	.07	.18	.22	.07	.12	.07	.09	0
mtsyn		8			1				0					0													53				27				
mtnon		134			189				10					67													149				288				

Figure S6. Synonymous (syn), non-synonymous (non) mutation counts for SHLP6, fraction conserved (cons) and fraction of synonymous mutations (fsyn), with vertebrate mitochondrial code synonymous (mtsyn) and non-synonymous (mtnon) counts.

Primates
nspec=242
>consensus

	G	F	T	T	S	M	L	D	Q	D	I	L	M	V	Q	P	L	L	r	V	R	L	F	N	D	x	S	P	T	w	S
	GGG	TTT	ACG	ACC	TCG	ATG	TTG	GAT	CAG	GAC	ATC	CTA	ATG	GTG	CAG	CCG	CTA	TTA	AGG	GTT	CGT	TTG	TTC	AAC	GAT	TAA	AGT	CCT	ACG	TGA	TCT
syn	0	0	0	0	0	n/a	0	0	79	11	0	9	0	0	32	0	2	21	0	62	0	0	0	0	0	0	1	0	0	0	0
non	55	13	0	0	0	0	0	0	1	0	68	83	21	0	46	97	0	86	132	6	4	0	0	1	72	0	21	87	6	6	8
cons	.79	.95	1.0	1.0	1.0	1.0	1.0	1.0	.99	1.0	.72	.66	.91	1.0	.81	.60	1.0	.64	.46	.98	.98	1.0	1.0	.99	.70	1.0	.91	.64	.98	.98	.97
fsyn	0	0	*	*	*	n/a	*	*	.99	1.0	0	.10	0	*	.41	0	1.0	.20	0	.91	0	*	*	0	0	*	.05	0	0	0	0
mtsyn						0					0		0						0		0					0				0	
mtnon						0					68		21						132		4					0				6	

Mammals
nspec=147
>consensus

	GGG	TTT	ACG	ACC	TCG	ATG	TTG	GAT	CAG	GAC	ATC	CCA	ATG	GTG	CAG	CCG	CTA	TTA	AAG	GTT	CGT	TTG	TTC	AAC	GAT	TAA	AGT	CCT	ACG	TGA	TCT
	G	F	T	T	S	M	L	D	Q	D	I	P	M	V	Q	P	L	L	K	V	R	L	F	N	D	x	S	P	T	w	S
syn	1	1	0	0	0	n/a	0	0	7	3	0	23	n/a	0	23	1	0	3	0	1	0	0	0	0	0	0	0	0	0	0	0
non	6	2	0	0	0	0	0	0	1	0	14	81	1	1	16	77	0	39	90	4	3	3	4	4	6	5	10	15	7	7	7
cons	.96	.99	1.0	1.0	1.0	1.0	1.0	1.0	.99	1.0	.90	.45	.91	.99	.90	.48	1.0	.73	.39	.97	.98	.98	.97	.97	.95	.97	.93	.90	.95	.95	.95
fsyn	.14	.33	*	*	*	n/a	*	*	.88	1.0	0	.22	n/a	0	.59	.01	*	.07	0	.20	0	0	0	0	0	0	0	0	0	0	0
mtsyn						0					0		0								0					0				0	
mtnon						0					14		1								3					5				7	

Vertebrates
nspec=348
>consensus

	GGG	TTT	ACG	ACC	TCG	ATG	TTG	GAT	CAG	GAC	ATC	CTA	ATG	GTG	CAG	CCG	CTA	TTA	AGG	GTT	CGT	TTG	TTC	AAC	GAT	TAA	AGT	CCT	ACG	TGA	TCT
	G	F	T	T	S	M	L	D	Q	D	I	L	M	V	Q	P	L	L	r	V	R	L	F	N	D	x	S	P	T	w	S
syn	0	0	1	0	1	n/a	0	2	8	10	0	21	n/a	0	46	2	8	30	0	1	0	0	0	0	0	0	9	2	0	0	0
non	59	12	0	0	0	0	0	0	5	13	52	91	39	15	37	99	3	56	143	10	13	3	4	4	18	6	94	89	77	78	78
cons	.83	.97	.99	1.0	1.0	1.0	1.0	1.0	.99	.96	.85	.74	.89	.96	.89	.72	.99	.84	.59	.97	.96	.99	.99	.99	.95	.98	.73	.74	.78	.78	.78
fsyn	0	0	1.0	0	1.0	*	*	1.0	.62	.43	0	.19	n/a	0	.55	.02	.73	.35	0	.09	0	0	0	0	0	0	.09	.02	0	0	0
mtsyn						0					0		0						0		0					0				0	
mtnon						0					52		39						143		13					6				78	

Figure S7. Top invertebrate Blastn hits to vertebrate consensus codon sequences of humanin, MOTS-c, SHLP2b, SHLP4, and SHLP6

Humanin

```
>vertebrate consensus
 M   A   K   R   G   L   N   C   L   L   L   P   I   S   E   I   D   L   P   V   Q   K   R   E   x
ATG GCA AAA CGA GGG CTT AAC TGT CTC TTA CTT CCA ATC AGT GAA ATT GAT CTC CCC GTG CAG AAG CGG GAA TAA
```

top invertebrate hit with blastn

Cybicola armatus isolate JCC01 large subunit ribosomal RNA gene, partial sequence; mitochondrial
Sequence ID: OL466820.1 Length: 520   Number of Matches: 1

```
Score           Expect  Identities      Gaps            Strand
85.7 bits(43)   3e-15   61/67(91%)      0/67(0%)        Plus/Plus
Query  9    ACGAGGGCTTAACTGTCTCTTACTTCCAATCAGTGAAATTGATCTCCCCGTGCAGAAGCG  68
            ||||||||||||||||||| |  ||| | |||||||||||||||||||||||||||||||
Sbjct  103  ACGAGGGCTTAACTGTCTCCTTTTTCAAGTCAGTGAAATTGATCTCCCCGTGCAGAAGCG  162

Query  69   GGAATAA  75
            || ||||
Sbjct  163  GGGATAA  169
```

MOTS-c

```
>vertebrate consensus
 M   r   W   E   E   M   G   Y   I   F   x   T   r   N   T   R   K
ATG AGA TGG GAA GAA ATG GGC TAC ATT TTC TAA ACT AGA AAC AAC CGA AAA
```

top invertebrate hit with blastn

Homarus americanus mitochondrial 12S ribosomal RNA
Sequence ID: X95491.1   Length: 294   Number of Matches: 1

```
Score           Expect  Identities      Gaps            Strand
50.1 bits(25)   9e-05   25/25(100%)     0/25(0%)        Plus/Plus
Query  9    GGAAGAAATGGGCTACATTTTCTAA  33
            |||||||||||||||||||||||||
Sbjct  87   GGAAGAAATGGGCTACATTTTCTAA  111
```

Figure 7. (continued)

```
SHLP2b
>vertebrate consensus
 V   A   A   F   r   P   T   M   V   V   V   L   F   T   L   L   L   r   L   L   P   S   V   x   K   L   V   P   L   x   L   P   L   L
GTG GCT GCT TTT AGG CCC ACT ATG GTT GTT GTT CTT TTT TAT CTC CTA CTT AAG TTG TTG TCT AGT TTT TAA AAG CTG GTT CCT CTT TAG TTA CCC TTT TTG

top invertebrate hit with blastn

Florometra serratissima mitochondrion, complete genome
Sequence ID: NC_001878.1  Length: 16005    Number of Matches: 1

Score            Expect   Identities       Gaps             Strand
40.1 bits(20)    0.23     35/40(88%)       0/40(0%) Plus/Plus
Query  1     GTGGCTGCTTTTAGGCCCACTATGGTTGTTGTTCTTTTTT  40
             |||||||||| |||||| |||||| || || |||||||||
Sbjct  4498  GTGGCTGCTTCTAGGCCTACTATGTTTATTATTCTTTTTT  4537


SHLP4
>vertebrate consensus
 M   L   E   A   M   F   L   V   N   r   R   G   L   C   L   P   S   S   F   Y   F   F   x   S   F   L   x
ATG CTA GAG GCG ATG TTT TTG GTA AAC AGG CGG GGT TTG TGT TTG CCG AGT TCC TTT TAC TTT TTT TAA TCT TTC CTT TAA

top invertebrate hit with blastn

Lysidice collaris voucher USNM1122617 16S ribosomal RNA gene, partial sequence; mitochondrial
Sequence ID: GQ478170.1   Length: 949      Number of Matches: 1

Score            Expect   Identities       Gaps             Strand
73.8 bits(37)    1e-11    46/49(94%)       0/49(0%) Plus/Minus
Query  8    AGGCGATGTTTTTGGTAAACAGGCGGGGTTTGTGTTTGCCGAGTTCCTT  56
            |||||||||||||| |||||||||| ||| |||||||||||||||||||
Sbjct  449  AGGCGATGTTTTTGTTAAACAGGCGAGGTATGTGTTTGCCGAGTTCCTT  401


SHLP6
>vertebrate consensus
 M   L   D   Q   D   I   L   M   V   Q   P   L   L   r   V   R   L   F   N   D   x
ATG TTG GAT CAG GAC ATC CTA ATG GTG CAG CCG CTA TTA AGG GTT CGT TTG TTC AAC GAT TAA

top invertebrate hit with blastn

Caligus olsoni voucher LACM:RW17.187.3455 large subunit ribosomal RNA gene, partial sequence; mitochondrial
Sequence ID: MN982271.1   Length: 774      Number of Matches: 1

Score            Expect   Identities       Gaps             Strand
125 bits(63)     3e-27    63/63(100%)      0/63(0%) Plus/Plus
Query  1    ATGTTGGATCAGGACATCCTAATGGTGCAGCCGCTATTAAGGGTTCGTTTGTTCAACGAT  60
            ||||||||||||||||||||||||||||||||||||||||||||||||||||||||||||
Sbjct  692  ATGTTGGATCAGGACATCCTAATGGTGCAGCCGCTATTAAGGGTTCGTTTGTTCAACGAT  751

Query  61   TAA  63
            |||
Sbjct  752  TAA  754
```

Figure S8. Comparison of human and murine SHLP2 and SHLP3 regions of MT-RNR2

SHLP2

Base alignment, Identity: 76.3%

```
>H sapiens  2090 TTAACTGTAGTCCAAAGAGGAACAGCTCTT-TGGACACTAGGAAAAAACCTTGTAGAGAGAGTAA--
                 |  | | ||| | ||||||| ||||||||| |||| ||  ||||||||||||  | |  || |||
>M musculus 1517 TAGATTATAGCCAAAAGAGGGACAGCTCTTCTGGA-AC--GGAAAAAACCTTTAATAGTGAATAATT

>H sapiens  ---AAAA-----TTTAACACCCAT 2170
               ||||     ||||||   |||
>M musculus AACAAAACAGCTTTTAAC---CAT 1616
```

Amino acid translations, Identity: 7.6%

```
>H sapiens  MGVKFFTLSTrFFPSVQrAVPLWTNSx
            |    |
>M musculus MVKSCFVNYSLLKVFSVPEELSLFWLxS
```

SHLP3

Base alignment, Identity: 68.1%

```
>H sapiens  1701 CTACCAGACAACCTTAGCCAAACCATTTACCCAAATAAA-GTATAGGCGATAGAAATTGAAACCT---
                  ||    |  |  | |  |||| |||||| || | |||| |||| || || |||||||   || |
>M musculus 1127 TTATACTATTATATAAATCAAAACATTTATCCTACTAAAAGTATTGGAGAAAGAAATTCGTACATCTA

>H sapiens  GGCGCAATAGATATAGTACCGCAAGGGAAAGATGAAAAATTATAACCAAGCAT 1817
            || || |||||  |||||||||||||||||||||||| | ||     ||| |
>M musculus GGAGCTATAGAACTAGTACCGCAAGGGAAAGATGAAAGACTAATTAAAAGTAA 1247
```

Amino acid translations, Identity: 34.2%, lacks start codon

```
>H sapiens  MLGYNFSSFPCGTISIAPGFNFYRLYFIWVNGLAKVVWx
             |    ||||||| ||||      |
>M musculus LLLISLSSFPCGTSSiAPrCTNFFLQYFxxDKCFDLYNSi
```

Figure S9. Codons and counts for MOTS-c residues 1-11 in 14 species analyzed in the earlier study.

```
MOTS-c base sequence for amino acid residues 1-11
>H sapiens
ATGAGGTGGCAAGAAATGGGCTACATTTTCTAC
>P troglodytes
ATGAGGTGGCAAGAAATGGGCTACATTTTCTAC
>P paniscus
ATGAGGCGGCAAGAAATGGGCTACATTTTCTAC
>P abelii
ATGGAGTGGCAAGAAATGGGCTACATTTTCTAC
>M musculus
ATGAAATGGGAAGAAATGGGCTACATTTTCTTA
>R norvegicus
ATGAAGCGGAAAGAAATGGGCTACATTTTCTTT
>H glaber
ATGAAATGGGAAGAAATGGGCTACATTTTCTTA
>C lupus
ATGAGGTGGGAAGAAATGGGCTACATTTTCTAC
>B taurus
ATGAAATGGGAAGAAATGGGCTACATTCTCTAC
>D rerio
ATGAAATGGGAAGAAATGGGCTACACTTTCTAC
>P leo
ATGAGATGGGAAGCAATGGGCTACATTTTCTAC
>U arctos
ATGGGGTGGGAAGAAATGGGCTACATTTTCTAT
>E caballus
ATGGGATGGAGAGAAATGGGCTACATTTTCTAC
>T truncatus
ATGGATTGGGAAGAAATGGGCTACATTTTCTAT
```

Codon counts for residues 4, 5, 7 and 9

Q4	CAA 4, GAA 8, AAA 1, AGA 1
E5	GAA 13, GCA 1
G7	GGC 14
I9	ATT 13, ACT 1
